# Burden of mosquito-borne diseases across rural versus urban areas in Cameroon between 2002 and 2021: prospective for community-oriented vector management approaches

**DOI:** 10.1186/s13071-023-05737-w

**Published:** 2023-04-19

**Authors:** Leo Dilane Alenou, Philippe Nwane, Lili Ranaise Mbakop, Michael Piameu, Wolfgang Ekoko, Stanislas Mandeng, Elisabeth Ngo Bikoy, Jean Claude Toto, Hugues Onguina, Josiane Etang

**Affiliations:** 1 Department of Biological Sciences, Faculty of Medicine and Pharmaceutical Sciences, University of Douala, P.O. Box 2701, Douala, Cameroon; 2 Malaria Research Laboratory, Yaoundé Research Institute (IRY), Organization for the Coordination of Endemic Diseases’ Control in Central Africa (OCEAC), P.O. Box 288, Yaoundé, Cameroon; 3 Department of Animal Biology and Physiology, Faculty of Sciences, University of Yaoundé I, P.O. Box 337, Yaoundé, Cameroon; 4 School of Health Sciences, Catholic University of Central Africa, P.O. Box 1110, Yaounde, Cameroon; 5 Department of Animal Biology and Physiology, University of Bamenda, Bambili, P.O. Box 39, Douala, Cameroon; 6 Department of Insect Biotechnology in Plant Protection, Institute for Insect Biotechnology, Faculty 09—Agricultural Sciences, Nutritional Sciences and Environmental Management, Justus-Liebig-University Gießen, Winchester Str. 2, 35394 Giessen, Germany

**Keywords:** Mosquitoes, Arbovirus diseases, *Plasmodium* spp., Urban and rural areas, Cameroon

## Abstract

**Background:**

Over the past two decades, Cameroon has recorded one of the highest rates of urban population growth in sub-Saharan Africa. It is estimated that more than 67% of Cameroon's urban population lives in slums, and the situation is far from improving as these neighbourhoods are growing at an annual rate of 5.5%. However, it is not known how this rapid and uncontrolled urbanization affects vector populations and disease transmission in urban versus rural areas. In this study, we analyse data from studies conducted on mosquito-borne diseases in Cameroon between 2002 and 2021 to determine the distribution of mosquito species and the prevalence of diseases they transmit with regards to urban areas versus rural areas.

**Methods:**

A search of various online databases, such as PubMed, Hinari, Google and Google Scholar, was conducted for relevant articles. A total of 85 publications/reports were identified and reviewed for entomological and epidemiological data from the ten regions of Cameroon.

**Results:**

Analysis of the findings from the reviewed articles revealed 10 diseases transmitted by mosquitoes to humans across the study regions. Most of these diseases were recorded in the Northwest Region, followed by the North, Far North and Eastern Regions. Data were collected from 37 urban and 28 rural sites. In the urban areas, dengue prevalence increased from 14.55% (95% confidence interval [CI] 5.2–23.9%) in 2002–2011 to 29.84% (95% CI 21–38.7%) in 2012–2021. In rural areas, diseases such as Lymphatic filariasis and Rift valley fever, which were not present in 2002–2011, appeared in 2012–2021, with a prevalence of 0.4% (95% CI 0.0– 2.4%) and 10% (95% CI 0.6–19.4%), respectively. Malaria prevalence remained the same in urban areas (67%; 95% CI 55.6–78.4%) between the two periods, while it significantly decreased in rural areas from 45.87% (95% CI 31.1–60.6%) in 2002–2011 to 39% (95% CI 23.7–54.3%) in the 2012–2021 period (*P = 0.04). Seventeen species of mosquitoes were identified as involved in the transmission of these diseases, of which 11 were involved in the transmission of malaria, five in the transmission of arboviruses and one in the transmission of malaria and lymphatic filariasis. The diversity of mosquito species was greater in rural areas than in urban areas during both periods. Of the articles reviewed for the 2012–2021 period, 56% reported the presence of *Anopheles gambiae* sensu lato in urban areas compared to 42% reported in 2002–2011. The presence of *Aedes aegypti* increased in urban areas in 2012–2021 but this species was absent in rural areas. Ownership of long-lasting insecticidal nets varied greatly from one setting to another.

**Conclusions:**

The current findings suggest that, in addition to malaria control strategies, vector-borne disease control approaches in Cameroon should include strategies against lymphatic filariasis and Rift Valley fever in rural areas, and against dengue and Zika viruses in urban areas.

**Graphical Abstract:**

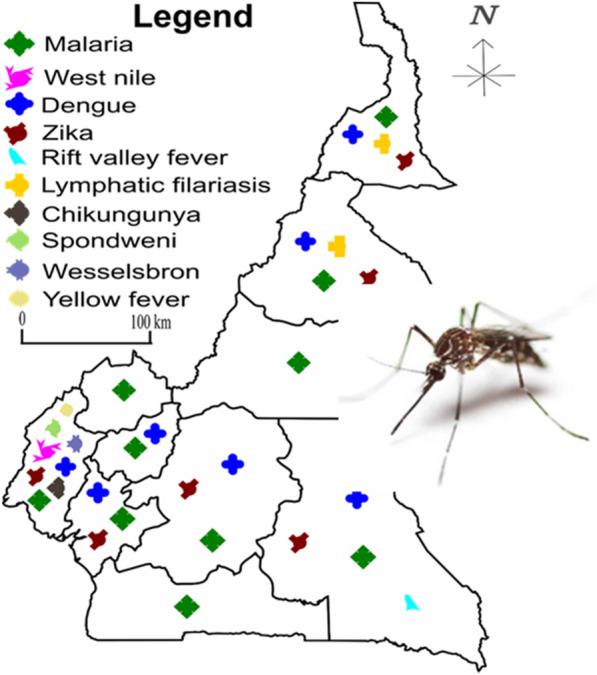

## Background

Mosquito-borne diseases (MBDs) such as malaria and arboviruses remain a major public health problem worldwide [1, 2]. In recent years, these diseases have become increasingly widespread, accounting for a significant proportion among the overall burden of infectious diseases. The WHO reported 2 million more cases of malaria in 2021 compared to 2020 [3]. Concerning arboviruses, recent estimates suggest that there are more than 3.5 billion people at risk of dengue virus (DENV) infection in > 100 countries and around 390 million DENV infections are reported each year, of which 100 million cause clinical symptoms [4]. The explosive 2015–2016 Zika virus (ZIKV) epidemic infected > 1 million people in 73 countries, inducing an increased incidence of microcephaly in newborns and requiring the WHO to declare ZIKV infection a public health emergency of international concern [5]. Chikungunya virus (CHIKV) epidemics also occur frequently around the world on all continents, with the number of cases reaching hundreds of thousands [6]. Overall, MBDs result in significant mortality and morbidity, causing millions of deaths, long-term disability and lifelong sequelae annually [7].

In the past, many of these diseases were largely confined to specific regions, especially in rural areas of the tropics and subtropics [8]. This situation is now being profoundly altered by many factors, including climate change, increased travel, migration and refugee movements, global trade, deforestation and unplanned urbanization [9]. Pathogens are not limited by national borders; local and international movements of people and goods can contribute to their rapid spread [10]. Increasing urbanization results in large and dense populations, which in turn increases the likelihood of transmission and outbreak of infectious diseases. In addition, climate change and human activities may expand the habitats of some vectors into new areas, exposing new populations to the diseases they transmit, with patterns and intensity fluctuating seasonally [11]. The diagnosis and prevention of and response to infectious disease threats are therefore critical to national and global health security.

In Cameroon, the situation is of concern because the country provides an ideal environment for mosquito-vector proliferation [12, 13]. One of the crucial elements in reducing the burden of vector-borne diseases is behavioural change of the affected populations, which can be achieved through education and increased public awareness, so that people know how to protect themselves and their communities from mosquitoes and other vectors, such as flies and ticks, among others. It is now well known that about 80% of "malaria" cases in Cameroon are treated by non-conventional means in many families. Indeed, there is a need to develop a health education programme in local communities and schools, with the aim to implement sanitation measures in neighbourhoods and teach people how to recognize early the febrile symptoms of common vector-borne diseases. The development of these programs will depend on the information collected at the community level regarding the MBDs and the bio-ecology of the vectors. However, little research has been conducted to improve understanding of the transmission of MBDs in municipalities. Furthermore, little information is available regarding changing epidemiological patterns and the real coverage of vector control interventions. Meanwhile, combining community knowledge on vector-borne diseases with diagnostic and surveillance strategies, may strengthen the effectiveness of prevention and control interventions.

The main objective of this review was to analyze the evolution of MBD transmission in urban versus rural areas in Cameroon over the past 20 years, in order to improve current knowledge on their differential burden and better inform, strengthen and guide community-based strategies against vector-borne diseases.

## Methods

### Literature search

An online search of online bibliographic databases, such as PubMed, Hinari, Google and Google Scholar, for scientific articles on MBDs in urban and rural areas of Cameroon was undertaken using different search terms. Search terms included a combination of key words, such as "urban/rural mosquito-borne pathogens", "urban/rural mosquito-borne diseases", "urban/rural mosquito-borne viruses", "urban/rural mosquito-borne bacteria", "mosquito fauna in urban or rural areas", mosquito genera (including "*Aedes*", "*Culex*", "*Mansonia*", "*Anopheles*"), "arboviruses", "malaria", "filariasis" and "Cameroon". In addition to the online bibliographic databases consulted, data were also extracted from reports and theses.

### Selection of articles

Articles selected were published between 2002, which is the year Cameroon developed the very first National Strategic Plan for Malaria Control (NSPMC) and restructured the National Malaria Control Programme to make it more operational and effective (NMCP), and 2021. The selected articles meet the following criteria: (i) data on Cameroon; (ii) no records repeated; (iii) prevalence data; (iv) entomological data; (v) diseases transmitted by mosquitoes; (vi) representative sampling; (vii) studies with field data collected between 2002 and 2021; and (viii) description of geographic or behavioural risk factors and changes in distribution of MBDs as a result of interventions.

### Study periods

The study was conducted between January and August 2022. It consisted of collecting data from published databases over two decades: (1) between 2002 and 2011; (ii) between 2012 and 2021.

### Data analysis

Selected articles were categorized based on their geographic location and type of MBDs. The information extracted from each selected study was recorded in a Microsoft Excel (Microsoft Corp., Redmond, WA, USA) spreadsheet for data analysis. This information included authors’ names, year of publication, study site, ecological profile (urban and rural), malaria transmission indices (entomological inoculation rate [EIR], human biting rate [HBR]), mosquito species, study period, prevalence of other MBDs and pathogen species. The EIR estimates and prevalences were not always available in selected papers in an adequate format for analysis. Consequently, a number of steps were undertaken to adjust data presentation (see Table 1). The prevalence of diseases in this paper was defined as the number of recorded infected people in a study population. As with the EIR data, a number of steps, as indicated in Table 2, were taken to adjust the presentation of prevalence data for different urban and rural areas. These same steps were taken to adjust data presentation of discarded prevalence. The Kruskal–Wallis and Mann–Whitney tests were used to compare the means of EIR estimates between urban and rural settings. The EIRs and parasite prevalence were also compared between the two periods study period, namely before 2011 and after 2011, because the studies conducted from 2012 to 2021 commonly used modern molecular and serological tools for mosquito processing and diagnosis, such as PCR and ELISA, while studies conducted in the previous decade (2002–2011) commonly used dissection and microscopy techniques and methods. Indeed, modern tests have a high sensitivity and specificity that improve diagnosis. Analyses were performed using R software version 4.1.0 (R Foundation for Statistical Computing, Vienna, Austria) and Epi Info 7 (Centers for Disease Control and Prevention, Atlanta, GA, USA)

**Table 1:** Action taken to adjust data presentation of entomological inoculation rates

Data available in the article	Action taken to adjust data presentation
When many EIRs were estimated for the same site (EIRs for districts within a city)	The average EIR from the area was estimated and used for analysis
When the EIR value was presented for 2 different periods in the same site	The highest value was considered
When indoor and outdoor EIRs were reported	The EIR means were used to estimate the total EIR from the area
When EIRs were presented as daily or monthly or seasonal EIRs	The annual EIR was estimated

*EIR* Entomological inoculation rates

**Table 2:** Action taken to adjust data presentation of prevalence

Data available in the article	Action taken to adjust data presentation
When the prevalence was estimated twice for the same site (prevalence for districts within a city)	The average prevalence from the area was estimated and used for analysis
When the prevalence value was presented for two different periods in the same site	The highest value was considered
When indoor and outdoor prevalence were reported	The prevalence means we used to have the total prevalence from the area
When the prevalence was presented as daily or monthly or seasonal prevalence	The annual prevalence was estimated

## Results

### Articles included in the study

**Fig. 1:**
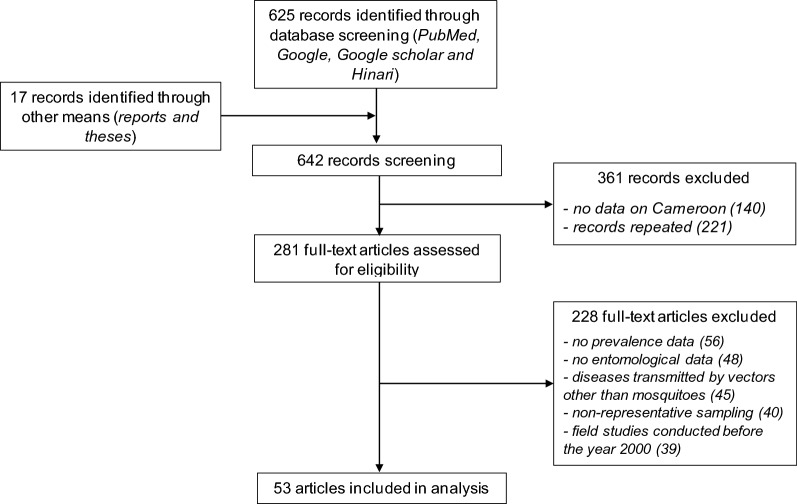
PRISMA chart showing the steps for the selection of articles. In total, 642 articles on mosquito-borne diseases published between 2002 and 2021 were identified from online bibliographic databases using different search terms and from other sources. After sorting the titles of publications and removing duplicates, 281 articles were assessed for eligibility and 288 were excluded. Ultimately, 53 articles were retained for analysis in this review

Our search identified a total of 642 records published between 2002 and 2021, among which 625 were identified from database searches and 17 were identified through other means (reports and theses). Ultimately, 53 articles were retained for analysis according to the above criteria. The selection procedure and validation of articles of interest areshown in Fig. 1. Among the 53 articles analysed (Table 3), 31.54% (47/149) focussed on the distribution of MBDs, 15.44% (23/149) reported the prevalence of MBDs, 18.12% (27/149) determined *Plasmodium* EIRs, 32.88% (49/149) showed the distribution of mosquito species involved in pathogen (arboviruses and parasites) transmission and 2.01% (3/149) addressed mosquito control interventions.

**Table 3:** Studies included in the review

Number of studies included in review by study period (decade)		Total studies included (*N* = 149)	Subject of study	References
2002–2011	2012–2021			
18	29	47	Distribution of mosquito-borne diseases	[14–51]
6	17	23	Prevalence of mosquito-borne diseases	[15, 22, 23, 27, 22, 23, 34, 36, 38, 41, 22, 23, 22, 23, 22, 23]
14	13	27	Distribution of *Plasmodium* EIR	[14, 16–25, 16–25, 33, 37, 16–25, 42, 45, 16–25]
19	30	49	Distribution of mosquito species responsible for transmission	[11, 14–34, 14–34, 14–34]
1	2	3	Mosquito control interventions	[62–64]

### Geographical distribution of the studies analysed on mosquito-borne diseases in Cameroon

**Fig. 2:**
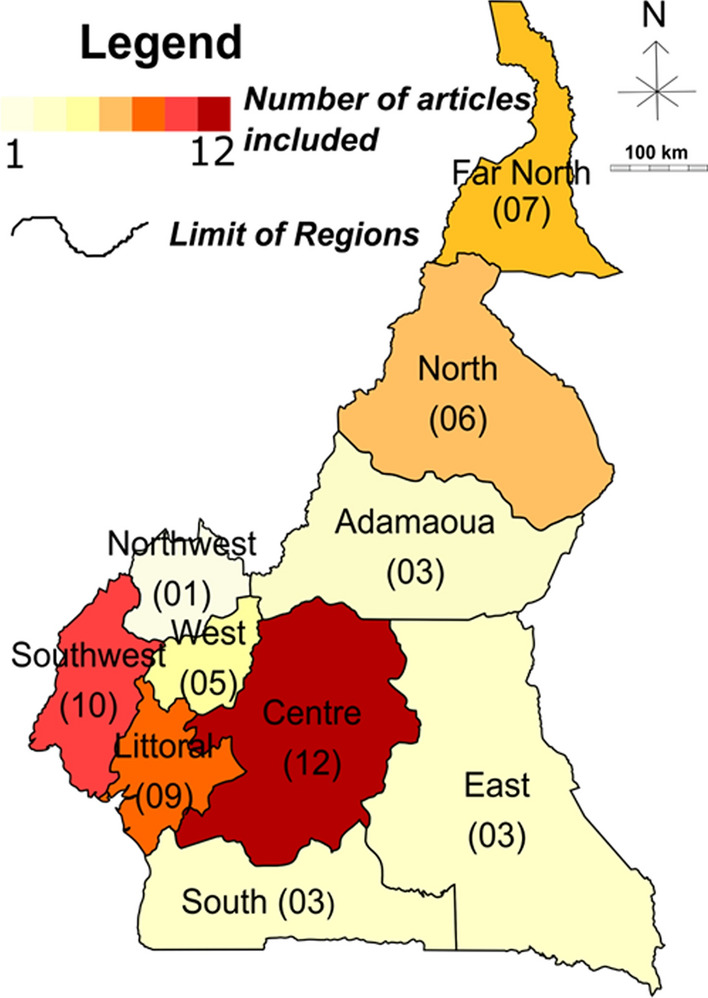
Geographic distribution of studies conducted in Cameroon related to mosquito-borne diseases between 2002 and 2021. The articles cover the whole country but are unevenly distributed among the 10 administrative regions. The number of items recorded per region are indicated by different colours (1–12). The black lines represent the boundaries between regions. Scale bars: 100 km

The 53 articles included in the review are representative of the whole country but are unevenly distributed across the ten administrative regions of Cameroon. The highest number of publications reported studies in the Centre and Southwest Regions, followed by the Littoral, Far North, North and West Regions. The lowest number of publications reported studies in the Adamaoua, East, South and Northwest Regions (Fig. 2).

**Fig. 3:**
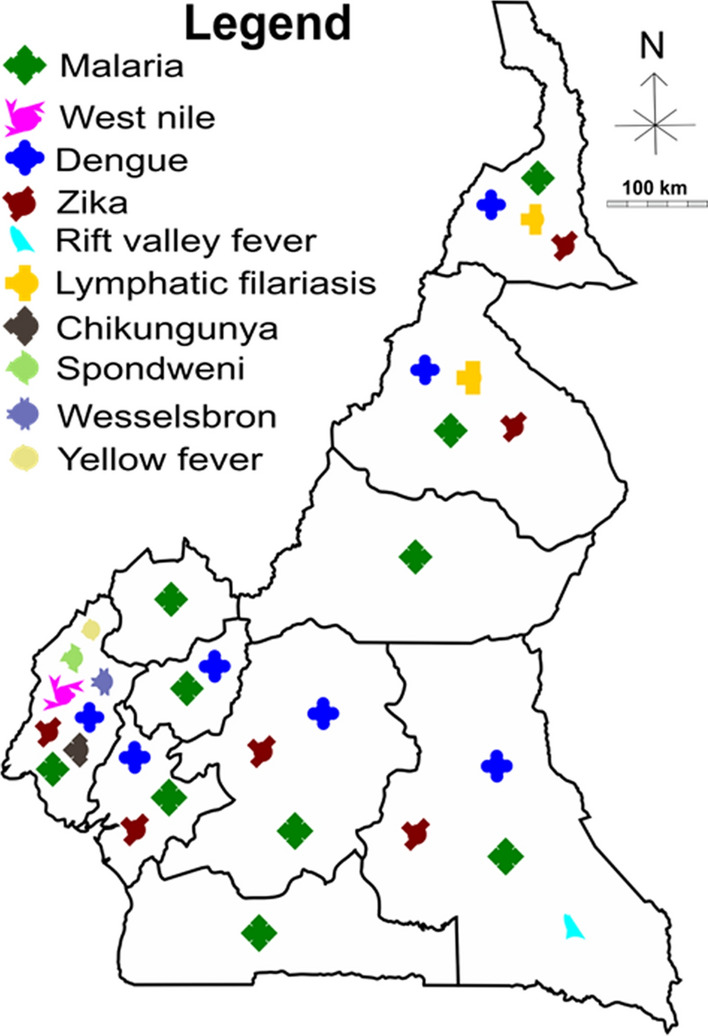
Map showing the localization of mosquito-borne infections across the 10 regions of Cameroon. Overall, malaria is present in all regions of the country. Chikungunya virus, West Nile virus, Spondweni virus, Wesselsbron disease virus and yellow fever virus were reported in the Southwest Region; dengue virus was reported in the Centre, East, Littoral, North, Far North, South and West Regions; lymphatic filariasis was reported in the North and Far North Regions; Rift Vally fever virus was reported in the Eastern Region; and Zika virus was reported in the Central, Eastern, Littoral, Northern, Far North and Southwestern Regions. The black lines represent the boundaries between regions. Scale bars: 100 km

The location of the study sites and their ecological features, the pathogens described and the references of the publications are given in Table 4. The pathogens belong to three groups, namely protozoans, arboviruses and parasitic worms. The group of protozoans is represented by the genus *Plasmodium*, the group of arboviruses by the genera *Flavivirus*, *Alphavirus* and *Phlebovirus* and the group of parasitic worms by *Wuchereria* spp. Overall, each region of the country recorded at least one case study on malaria within the past 20 years. CHIKV, West Nile virus (WNV), Spondweni virus (SPOV), Wesselsbron (WSL) virus and Yellow fever virus (YFV) were reported in the Southwest Region. DENV was recorded in the Centre, East, Littoral, North, Far North, South and West Regions. Lymphatic filariasis (LF) was reported in the North and Far North Regions, Rift Valley Fever (RVF) virus was recorded in the East Region and ZIKV was recorded in the Centre, East, Littoral, North, Far North and Southwest Regions (Fig. 3).

**Table 4:** Study sites and their ecological features

Study sites	Geographical coordinates	Ecological features	Diseases	Genus	References
Akonolinga	03°54’N, 12°31’E	Rural	Malaria	*Plasmodium*	[19, 47]
Ayos	04°00’N, 12°19’E	Rural	Malaria	*Plasmodium*	[16]
Kombo	04°05’N, 12°24’E	Rural	Malaria	*Plasmodium*	[14]
Mbalmayo	03°46’N, 12°15’E	Urban	Malaria	*Plasmodium*	[21]
Mvoua	04°04’N, 11°25’E	Rural	Malaria	*Plasmodium*	[42]
Ntui	04°26’N, 11°37’E	Urban	Malaria	*Plasmodium*	[15]
			Dengue	*Flavivirus*	
Bafia	04°45’N, 11°14’E	Urban	Malaria	*Plasmodium*	[15]
Ndelle	03°51’N, 11°30’E	Urban	Malaria	*Plasmodium*	[14]
Olama	05°56’N, 10°32’E	Urban	Malaria	*Plasmodium*	[21, 23]
Yaounde	03°24’N, 11°18’E	Rural	Malaria	*Plasmodium*	[15, 19, 28, 29, 43, 54]
			Dengue	*Flavivirus*	
			Zika	*Flavivirus*	
Bertoua	04°34’N, 13°41’E	Urban	Malaria	*Plasmodium*	[26, 34]
			Dengue	*Flavivirus*	
			Zika	*Flavivirus*	
Lomie	03°10’N, 13°37’E	Rural	Malaria	*Plasmodium*	[56]
Messok	03°05’N, 14°03’E	Rural	RVF	*Phlebovirus*	[56]
Mindourou	03°10’N, 13°37’E	Rural	RVF	*Phlebovirus*	[56]
Yabassi	04°29’N, 09°58’E	Urban	Malaria	*Plasmodium*	[17]
Loum	03°46’N, 12°15’E	Urban	Malaria	*Plasmodium*	[47]
Douala	04°03’N, 09°42’E	Urban	Dengue	*Flavivirus*	[15, 18, 41, 54, 55, 58]
			Malaria	*Plasmodium*	
			Zika	*Flavivirus*	
Manoka	03°47’N, 09°39’E	Rural	Malaria	*Plasmodium*	[27]
Youpwe	04°00’N, 09°42’E	Urban	Malaria	*Plasmodium*	[27]
Ndogbassi	03°48’N, 10°08’E	Urban	Malaria	*Plasmodium*	[65]
Njombe	04°34’N, 09°39’E	Urban	Malaria	*Plasmodium*	[39]
Bonandam	04°35’N, 09°40’E	Rural	Malaria	*Plasmodium*	[39]
Edéa	03°48’N, 10°08’E	Urban	Dengue	*Flavivirus*	[15]
			Malaria	*Plasmodium*	
Douvar	13°38’N, 10°57’E	Urban	Malaria	*Plasmodium*	[22]
Maroua	10°35’N, 14°19’E	Urban	Malaria	*Plasmodium*	[48, 54]
			Zika	*Flavivirus*	
Mokolo	13°80’N, 10°75’E	Rural	Malaria	*Plasmodium*	[37, 57]
			LF	*Brugia*	
Godola	10°70’N, 14°25’E	Rural	Malaria	*Plasmodium*	[37]
Maga	10°50’N, 14°56’E	Rural	Malaria	*Plasmodium*	[26]
Kaelé	10°06’N, 14°27’E	Urban	Malaria	*Plasmodium*	[15]
			Dengue	*Flavivirus*	
Tibati	06°27’N, 12°37’E	Rural	Malaria	*Plasmodium*	[32]
Ngaoundere	07°19’N, 13°35’E	Urban	Zika	*Flavivirus*	[54]
Bankim	06°00’N, 11°40’E	Urban	Malaria	*Plasmodium*	[15]
			Dengue	*Flavivirus*	
Gounougou	09°05’N, 13°40’E	Rural	Malaria	*Plasmodium*	[22]
Garoua	09°18’N, 13°24’E	Urban	Malaria	*Plasmodium*	[30, 49, 54, 55]
			Dengue	*Flavivirus*	
			Zika	*Flavivirus*	
Pitoa	09°21’N, 13°31’E	Urban	Malaria	*Plasmodium*	[30, 49]
Mayo Oulo	09°46’N, 13°44’E	Urban	Malaria	*Plasmodium*	[30, 49]
Rey-Bouba	08°40’N, 14°10’E	Rural	FL	*Wuchereria*	[57]
Niete	02°43’N, 10°04’E	Rural	Malaria	*Plasmodium*	[25]
Ebolowa	02°54’N, 11°09’E	Urban	Malaria	*Plasmodium*	[33]
Nyabessan	02°80’N, 10°25’E	Rural	Malaria	*Plasmodium*	[40]
Tiko	04°07’N, 09°36’E	Rural	Spondweni	*Flavivirus*	[20, 24, 31, 53]
			Malaria	*Plasmodium*	
			Dengue	*Flavivirus*	
			Chikungunya	*Flavivirus*	
			West Nile	*Flavivirus*	
			Yellow fever	*Flavivirus*	
Limbe	04°02’N, 09°11’E	Urban	Spondweni	*Flavivirus*	[24, 53]
			Malaria	*Plasmodium*	
			Dengue	*Flavivirus*	
			Chikungunya	*Flavivirus*	
			West Nile	*Flavivirus*	
			Yellow fever	*Flavivirus*	
Idenau	04°01’N, 09°03’E	Urban	Malaria	*Plasmodium*	[24]
Fako	04°16’N, 09°16’E	Urban	Wesselsbron	*Flavivirus*	[52]
			Dengue	*Flavivirus*	
			Yellow fever	*Flavivirus*	
			Zika	*Flavivirus*	
Likoko Membea	04°08’N, 09°04’E	Urban	Malaria	*Plasmodium*	[31]
Meanja	04°18’N, 09°24’E	Urban	Malaria	*Plasmodium*	[31]
Mutengene	04°05’N, 09°18’E	Urban	Malaria	*Plasmodium*	[31, 36]
Debundscha	04°04’N, 09°04’E	Urban	Malaria	*Plasmodium*	[31]
Bomaka	04°09’N, 09°18’E	Rural	Malaria	*Plasmodium*	[38]
Molyko	04°09’N, 09°14’E	Urban	Malaria	*Plasmodium*	[38]
Buea	04°09’N, 09°13’E	Urban	Chikungunya	*Flavivirus*	[53]
			Dengue	*Flavivirus*	
			Yellow fever	*Flavivirus*	
Muyuka	04°10’N, 09°25’E	Urban	Chikungunya	*Flavivirus*	[53]
			Dengue	*Flavivirus*	
			Yellow fever	*Flavivirus*	
			Spondweni	*Flavivirus*	
			West Nile	*Flavivirus*	
Nkongho-mbeng	05°19’N, 09°40’E	Rural	Malaria	*Plasmodium*	[46]
Bolifamba	04°08’N, 09°18’E	Urban	Malaria	*Plasmodium*	[61]
Dibanda	04°06’N, 09°18’E	Rural	Malaria	*Plasmodium*	[51]
Mamfe	05°46’N, 09°17’E	Rural	Malaria	*Plasmodium*	[20]
Santchou	05°15’N, 09°50’E	Rural	Malaria	*Plasmodium*	[20, 50]
Ndop	05°56’N, 10°32’E	Rural	Malaria	*Plasmodium*	[20]
Foumbot	05°30’N, 10°37’E	Urban	Malaria	*Plasmodium*	[44]
Bamendjou	05°23’N, 10°18’E	Rural	Malaria	*Plasmodium*	[44]
Tonga	04°58’N, 10°41’E	Rural	Malaria	*Plasmodium*	[45]
Bangangte	05°09’N, 10°31’E	Urban	Malaria	*Plasmodium*	[15]
			Dengue	*Flavivirus*	
Foumban	05°43’N, 10°55’E	Urban	Malaria	*Plasmodium*	[15]
			Dengue	*Flavivirus*	
Dschang	05°23’N, 10°10’E	Urban	Malaria	*Plasmodium*	[15, 50]
			Dengue	*Flavivirus*	
Santchou	05°15’N, 09°50’E	Rural	Malaria	*Plasmodium*	[20, 50]
Bamenda	05°56’N, 10°12’E	Urban	Malaria	*Plasmodium*	[66]

### Prevalence of mosquito-borne diseases

**Fig. 4:**
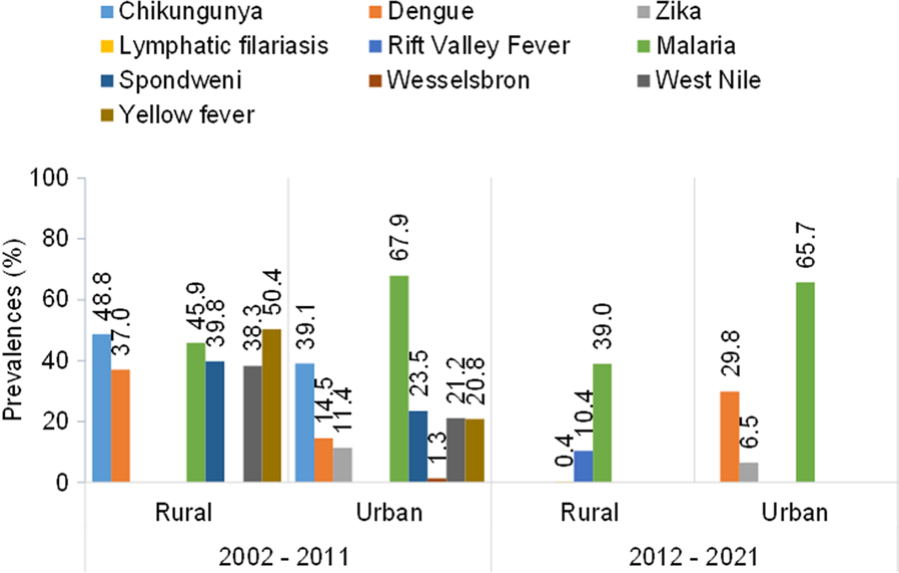
Prevalence of infections recorded between the years 2002 and 2021 in urban and rural areas. Data from urban and rural areas were calculated and expressed as a percentage (values above columns)

**Fig. 5:**
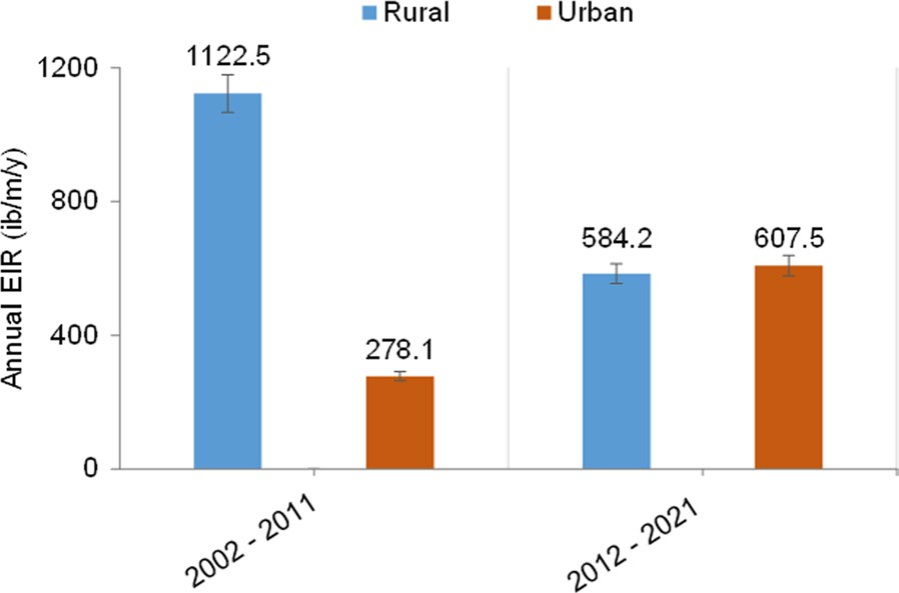
Distribution of *Plasmodium* EIR between rural and urban areas. Errors bars represent 95% confidence interval

The reported prevalences of CHIKV, SPOV, WSL, WNV and YFV ranged from 38% to 51% and from %1.3 to 40% in rural and urban areas, respectively, during the period 2002–2011. The diseases caused by these viruses completely disappeared after 2011. DENV, which had a prevalence of 37.07% (95% confidence interal [CI] 24.3–49.8%) in rural areas between 2002 and 2011, was no longer recorded in the same areas from 2012 to 2021. Conversely, in urban areas, the prevalence of this disease increased from 14.55% (95% CI 5.2–23.9%) in 2002–2011 to 29.84% (95% CI 21–38.7%) in 2012–2021. In rural areas, neglected tropical MBDs, such as LF and RVF, were not recorded in 2002–2011 while they appeared in 2012–2021, with prevalences ranging from 0.4% (95% CI 0.0–2.3%]) to 10% (95% CI 0.6–19.4%]), respectively. The prevalence of malaria remained the same in urban areas (67%) during the two periods 2002–2011 and 2012–2021; however, this prevalence may have decreased in rural areas from 45.87% (95% CI 31.1–60.6%) in 2002–2011 to 39% (95% CI 23.7–54.3%) in 2012–2021 (*P* = 0.91) (Fig. 4). Comparisons of the EIR estimates between urban and rural areas during the periods 2002–2011 and 2012–2021 revealed that malaria transmission has significantly decreased in rural areas (*P = 0.04), while it has significantly increased in urban areas (*P = 0.02) (Fig. 5).

### Distribution of mosquito species involved in vector-borne disease transmission during the last two decades

**Fig. 6:**
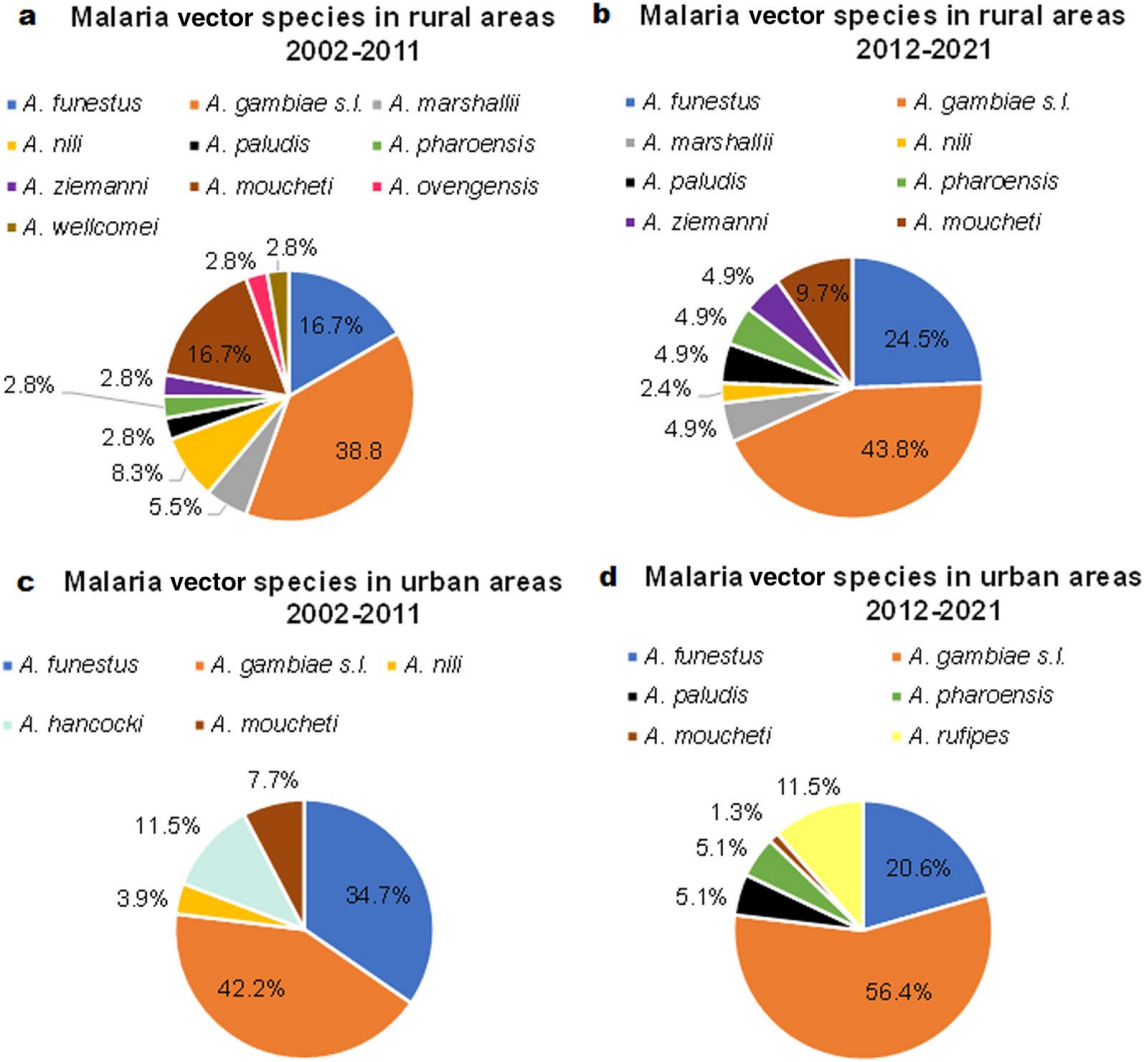
Distribution of malaria vector species between rural and urban areas. Seventeen species of mosquitoes involved in vector-borne disease transmission were identified in the studies published between 2002 and 2021 included in this review. **a**–**d** Proportion of studies reporting the presence of malaria vector species in rural areas in 2002–2011 (**a**), in rural areas in 2012–2021 (**b**), in urban areas in 2002–2011 (**c**) and in urban areas in 2012–2021 (**d**)

**Fig. 7:**
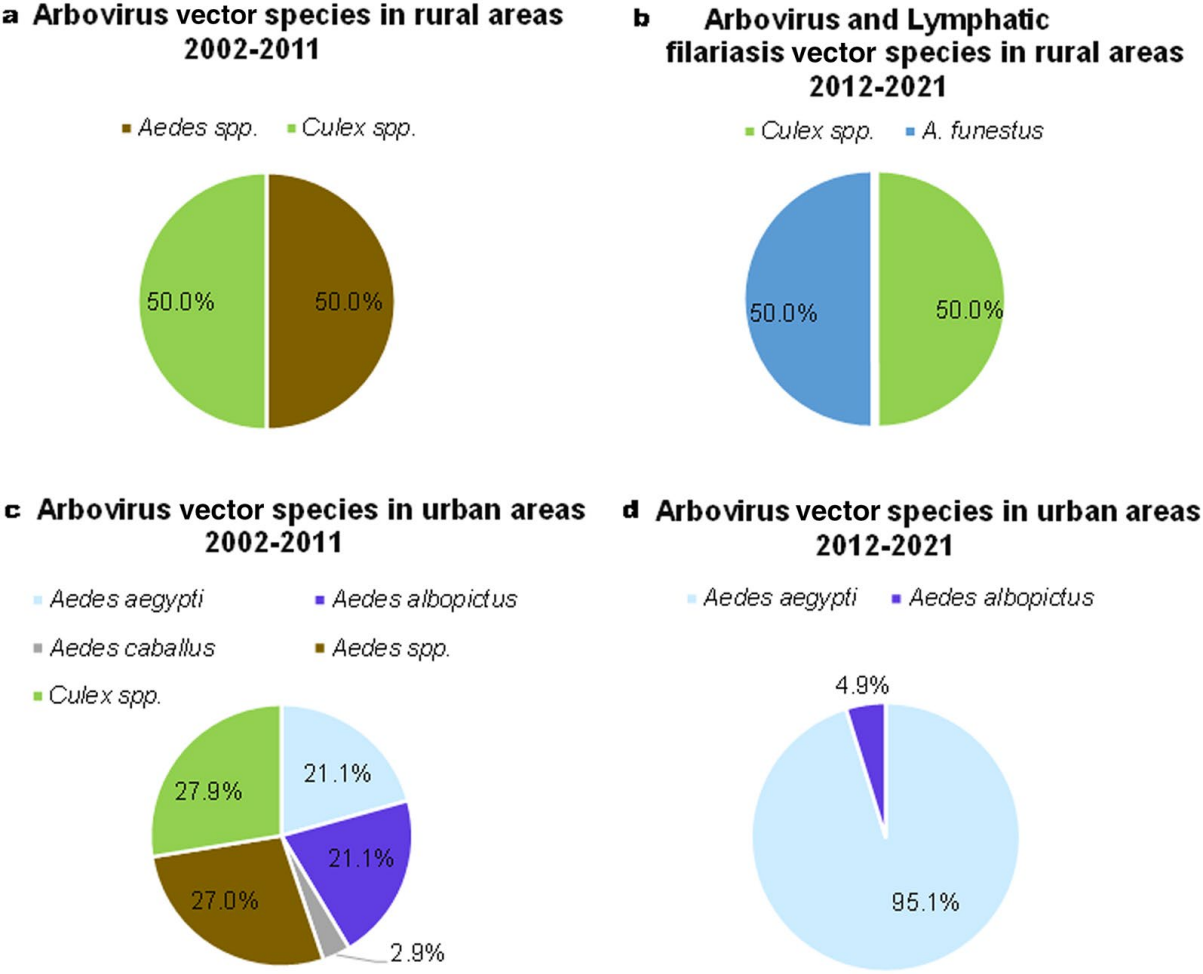
Distribution of arbovirus and lymphatic filariasis vector species between rural and urban areas. **a** Proportion of studies reporting the presence of arbovirus vector species in rural areas in 2002–2011,** b** proportion of studies reporting the presence of arbovirus and lymphatic filariasis vector species in rural areas in 2012–2021, **c** proportion of studies reporting the presence of arbovirus vector species in urban areas in 2002–2011, **d** proportion of studies reporting the presence of arbovirus vector species in urban areas in 2012–2021

Seventeen species of mosquitoes involved in vector-borne disease transmission have been recorded in published papers between 2002 and 2021. Of these 17 species, 11 were involved in the transmission of malaria, five in the transmission of arboviruses and one in the transmission of both malaria and LF. The diversity of mosquito species was greater in rural areas than in urban areas during both periods (Fig. 6a, b). Two major malaria vectors (*Anopheles gambiae* and *Anopheles funestus*) were more prevalent in urban areas than in rural areas (Fig. 6c, d). Of the articles included in this review that were published within the 2012–2021 period, 56% reported the presence of *A. gambiae* sensu lato in urban areas; in the 2002–2011 period, this was 42% of papers. The presence of *Aedes aegypti* increased in urban areas in 2012–2021 while this species was absent from rural areas; in the latter areas, *Aedes* spp. (50%) and *Culex* spp. (50%) were the most important arboviruses vectors for the 2002–2011 decade (Fig. 7). Of the papers published between 2012 and 2021, 50% reported *A. funestus* as the vector for LF in rural areas (Fig. 7b). Arbovirus vectors showed a significant decrease in species diversity in urban areas (Fig. 7c, d). *Aedes aegypti* (95%) and *Aedes albopictus* (5%) were the most prevalent species in urban areas during the 2012–2021 decade.

### Mosquito control interventions

**Fig. 8:**
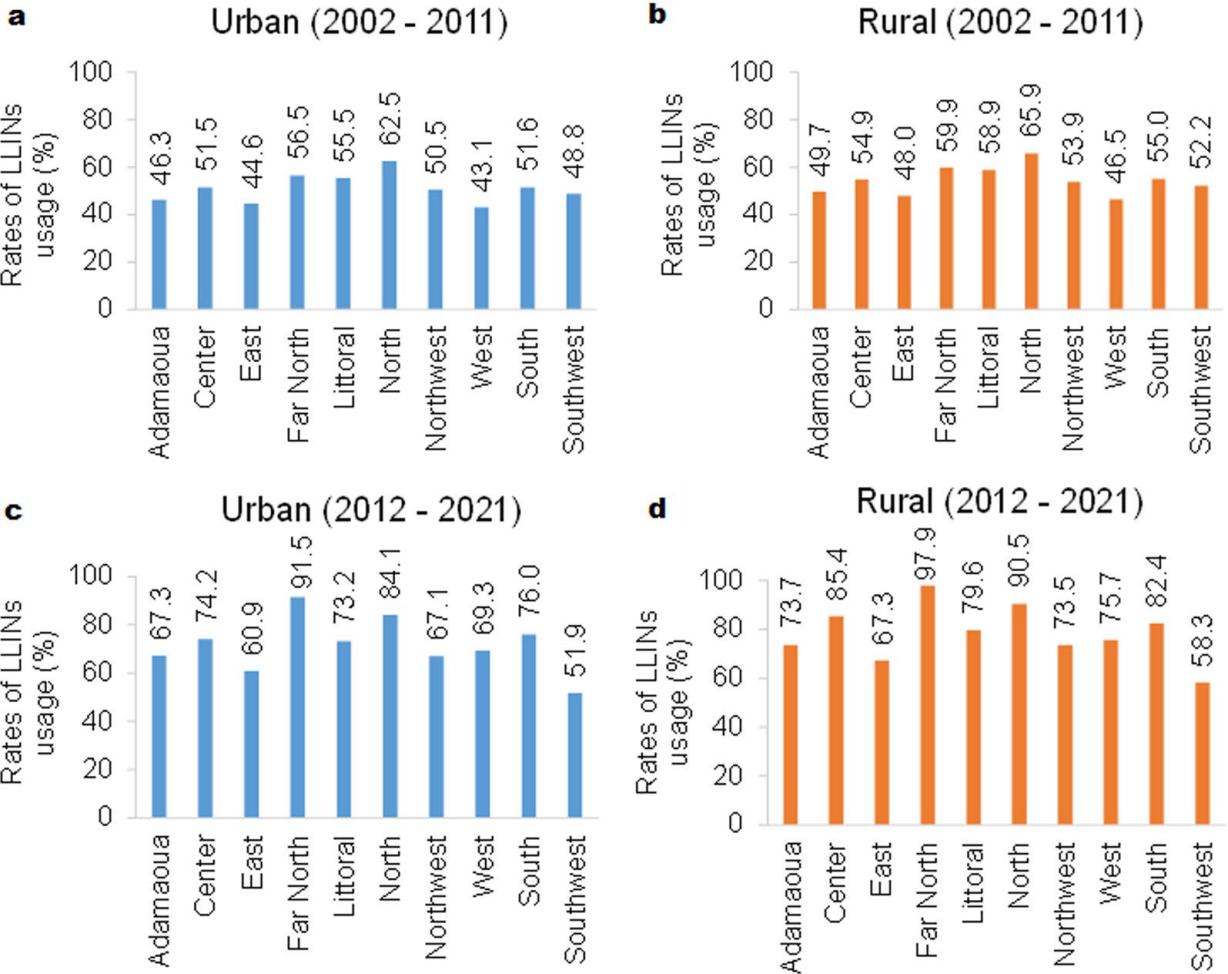
Rates of LLINs use between 2002 and 2021. The rate of LLIN use is higher in rural areas in both periods (2002–2011 and 2012–2021). **a**–**d** The rates of LLIN use by households in urban areas between 2002 and 2011 (**a**), by households in rural areas between 2002 and 2011 (**b**), by households in urban areas between 2012 and 2021 (**c**) and by households in rural areas between 2012 and 2021 (**d**). LLINs, Long-lasting insecticidal nets

Vector control made significant progress between 2002 and 2021, particularly for malaria control [67, 68]. Since 2000, Cameroon has benefitted from the support of various international partners to implement malaria control interventions [68]. Environmental management has been improved through major development projects (water drainage, filling of ponds) and maintenance of canals and banks, elimination of aquatic plants and management of water levels on a regular basis, with the overall aim to limit the development of mosquito larvae. This mechanical control also includes the elimination of all containers known as suitable breeding sites for mosquito larvae. This latter strategy is of considerable importance in the fight against *Aedes*, the vectors of arboviruses, since many of their breeding sites are created by humans. Insecticide-based mosquito control is still very important in vector control strategies, especially in areas with non-removable breeding sites and/or to target adult mosquitoes. Free distribution of insecticide-treated nets (ITNs) and long-lasting insecticidal nets (LLINs) is a major component of malaria vector control in Cameroon [69], in both rural and urban areas. Therefore, no significant difference in LLIN ownership was recorded in this study between urban and rural areas. However, an increase in LLIN ownership was observed over the time covered by this study in all regions, although it greatly varied from one region to another. The percentage of households owning at least one LLIN increased from 51.09% (Fig. 8a) to 54.49% (Fig. 8c) in urban areas and from 54.49% (Fig. 8b) to 78.43% (Fig. 8d) in rural areas between 2002–2011 and 2012–2021, respectively. During the 2002–2011 decade and for both urban and rural areas, the West (urban: 43.1%; rural: 46.5%), East (urban: 44.6%; rural: 48%) and Adamaoua (urban: 46.3%; rural: 49.7%) Regions had the lowest proportion of households owning at least one net. Net ownership was somewhat higher in the North (urban: 62.5%; rural: 65.9%) and Far North (urban: 56.5%; rural: 59.9%) Regions.

However, for the 2012–2021 decade, the Southwest (urban: 51.9%; rural: 58.3%) and East (urban: 60.9%; rural: 67.6%) Regions had the lowest percentage of households owning at least one LLIN. Other vector control interventions based on larviciding and the drainage of watercourses in the framework of the YSDP (Yaoundé Sanitation and Development Programme) have been carried out in the city of Yaoundé during the 2011–2022 decade [70].

## Discussion

The publications and other reports included in this review provided data on the evolution of MBD transmission in urban and rural areas over the past 20 years in Cameroon. Urbanization is increasingly blamed for influencing the epidemiology and evolution of vector-borne diseases in Cameroon [71, 73]. According to reports drawn up by the Monitoring Committee of the 4th General Census of Population and Housing in 2015, the rate of urbanization in Cameroon is increasing in an exponential manner, rising from 48.8% in 2002 to nearly 60% in 2021. Conversely, the growth rate of the rural population has dropped from 1.6% in 2002 to 1.2% in 2021 [72]. Nevertheless, the urbanization that has taken place over the last two decades has been punctuated by progress in social development, with the aim to improve hygiene and public health conditions of the populations. The decades prior to 2011 were marked by the creation and development of disease control programmes, including those against vector-borne diseases such as malaria (NMCP in 1997), onchocerciasis and LF, human and animal trypanosomiasis and schistosomiasis, all in 2003. It was also a time of increasing awareness of vector-borne diseases, with the introduction of a number of new diseases. The distribution of MBD studies included in this review, across the country, is very heterogeneous; in addition to the eco-climatic and phytogeographic characteristics which are specific to each of the 10 regions of Cameroon, the choice of the study sites in rural or urban areas depends on many other factors, including the availability of financial resources requested to collect samples in the field and to preserve and analyse the collected samples in the laboratory. Many research studies are conducted in the Central, Littoral and Southwest Regions where research institutions and universities are located.

Among the MBDs identified in this review, malaria and DENV are the most predominant, as they are distributed in almost all regions of Cameroon. The zonal distribution of other mosquito-transmitted diseases, such as YFV, LF and WNV, for example, reflect rather the lack of studies. Mosquito-borne diseases, except malaria and arboviral diseases, were reported more frequently in rural and urban areas in the 2002–2011 decade than in the 2012–2021 period. During the second period, the number of cases of urban malaria was stable while those of rural malaria decreased, but the number of DENV cases increased in urban areas. This observation reflects the country's progress over the 2012–2021 decade in the area of public health, particularly in terms of chemoprophylaxis through vaccination (e.g. the case of the yellow fever vaccine), vector control (e.g. distribution of mosquito nets) and diagnosis and case management in urban and rural areas.

Despite significant improvements in health conditions for human populations, malaria and DENV have remained endemic in rural and urban areas over this last decade. Although several strategies are deployed in the fight against malaria, this parasitic disease persists in urban and rural areas with high EIRs (approx. 600 ib/man/year) in urban areas. LLINs appear to be the most widely used preventive tool by urban and rural populations. This rate of use, although not satisfactory, is linked to the massive and free distribution of LLINs during campaigns conducted by the public authorities and their development partners with a view to providing every household in Cameroon with LLINs [74]. However, many studies have shown that this tool only has a real impact in an environment when it is used on a large scale at the community level and, therefore, the effect of LLINs depends on the habits of the resident population [75]. Unfortunately, in both urban and rural areas in most administrative regions, with the exception of the North and Far North Regions, usage rates are far from the 80% recommended by WHO for effective LLIN action [75]. Indeed, in most localities in Cameroon, a significant proportion of the population uses LLINs for other purposes (fishing and agriculture) [75], while others do not use LLINs for various reasons such as heat, allergies, the feeling of being locked up in a coffin and the preference not to use them. In addition, the poverty of the population leads some people to self-medicate and to use street medicines to treat themselves. This situation may explain the persistence of malaria cases in urban and rural areas. The government should undertake actions to assess knowledge, attitudes and practices regarding malaria treatment and prevention in order to provide sustainable solutions.

Many of the mosquito species involved in disease transmission show a preference for rural areas, but those responsible for malaria and DENV transmission, including *A. gambiae* and *A. funestus* complexes and *Aedes* are also prevalent in urban areas. Species belonging to the *A. gambiae* and *A. funestus* complexes are associated with human activities and are the main vectors of malaria in Cameroon and in other African countries [11]. The high frequencies of insecticide resistance mutations and genes in members of these species’ complexes coupled with their vectorial capacities and competences make them the most abundant and widespread mosquitoes in rural and urban areas. The increase in population density of these anopheline species through resistance to control products is further facilitated by the insecticide pressure acting on aquatic stages developing in agricultural settings, including coffee, sugar cane, cotton and rice fields [70], market gardening areas [76] and timber yards [59]. This insecticide pressure has progressively increased with the intensification of vector control through the distribution of ITNs to pregnant women and children aged < 5 years in the 2002–2011 decade and LLINs in the 2012–2021 decade.

## Conclusion

This study provides an update on MBDs in urban versus rural areas in Cameroon over the past two decades. While the prevalence of MBDs has decreased in rural areas between the two compared periods, MBDs in urban areas have increased and are likely to increase with continued unplanned urbanization. To halt this trend in disease burden, concerted actions must be taken quickly at various levels to improve prevention and control, through case detection and management and through vector control. In addition to malaria control strategies, vector-borne disease control approaches in Cameroon should include strategies against LF and RVF in rural areas, or against dengue in urban areas. Future research efforts should prioritize a better understanding of how the different interventions can be integrated and adapted to local context either in urban or in rural areas.

## Abbreviations

CHIKV: Chikungunya virus
DENV: Dengue virus
EIRs: Entomological inoculation rate
ITNs: Insecticide-treated nets
LF: Lymphatic filariasis
LLINs: Long-lasting insecticidal nets
MBDs: Mosquito-borne diseases
RVF: Rift Valley fever
SPOV: Spondweni virus
WNV: West Nile virus
WSL: Wesselsbron disease
YFV: Yellow fever virus
ZIKV: Zika virus
